# Exploring the antimicrobial, antioxidant, anticancer, biocompatibility, and larvicidal activities of selenium nanoparticles fabricated by endophytic fungal strain *Penicillium verhagenii*

**DOI:** 10.1038/s41598-023-35360-9

**Published:** 2023-06-03

**Authors:** Abdel-Rahman A. Nassar, Ahmed M. Eid, Hossam M. Atta, Wageih S. El Naghy, Amr Fouda

**Affiliations:** 1 Tanta Universal Teaching Hospital, Tanta University, Tanta, Egypt; 2 Botany and Microbiology Department, Faculty of Science, Al-Azhar University, Nasr City, Cairo, 11884 Egypt; 3 Department of Medical Microbiology and Immunology, Faculty of Medicine, Tanta University, Tanta, Egypt

**Keywords:** Biotechnology, Cancer, Microbiology, Nanoscience and technology

## Abstract

Herein, four endophytic fungal strains living in healthy roots of garlic were used to produce selenium nanoparticles (Se-NPs) via green synthesis. *Penicillium verhagenii* was found to be the most efficient Se-NPs producer with a ruby red color that showed maximum surface plasmon resonance at 270 nm. The as-formed Se-NPs were crystalline, spherical, and well-arranged without aggregation, and ranged from 25 to 75 nm in size with a zeta potential value of −32 mV, indicating high stability. Concentration-dependent biomedical activities of the *P. verhagenii*-based Se-NPs were observed, including promising antimicrobial activity against different pathogens (*Escherichia coli*, *Pseudomonas aeruginosa*, *Bacillus subtilis*, *Staphylococcus aureus, Candida albicans, C. glabrata, C. tropicalis,* and *C. parapsilosis*) with minimum inhibitory concentration (MIC) of 12.5–100 µg mL^–1^. The biosynthesized Se-NPs showed high antioxidant activity with DPPH-scavenging percentages of 86.8 ± 0.6% at a concentration of 1000 µg mL^–1^ and decreased to 19.3 ± 4.5% at 1.95 µg mL^–1^. Interestingly, the Se-NPs also showed anticancer activity against PC3 and MCF7 cell lines with IC_50_ of 225.7 ± 3.6 and 283.8 ± 7.5 µg mL^–1^, respectively while it is remaining biocompatible with normal WI38 and Vero cell lines. Additionally, the green synthesized Se-NPs were effective against instar larvae of a medical insect, *Aedes albopictus* with maximum mortality of 85.1 ± 3.1, 67.2 ± 1.2, 62.10 ± 1.4, and 51.0 ± 1.0% at a concentration of 50 µg mL^–1^ for I, II, III, and IV-instar larva, respectively. These data highlight the efficacy of endophytic fungal strains for cost-effective and eco-friendly Se-NPs synthesis with different applications.

## Introduction

Selenium is an important trace element for the flourishing of microorganisms and an essential micronutrient for animal and human health. Despite its beneficial properties, it is governed by a narrow therapeutic window. Excessive intake of organic and inorganic selenium compounds may lead to toxicity. Fortunately, selenium nanoparticles (Se-NPs) are less toxic than organic and inorganic selenium compounds^1^. Nanomaterials (1–100 nm) are unique in many chemical and physical properties that distinguish them from their counterparts in bulk materials. These materials have been adopted and applied for agricultural, environmental, and medical fields^2,3^. Additionally, the biosynthesized Se-NPs are distinguished from those manufactured by chemical and physical methods in that they are more compatible with human tissues and organs^4^. The biological synthesis pathways have been explored using plants and fungi to produce nanoparticles in sustainable and environmentally safe manner^5,6^. Endophytes are microorganisms including fungi, bacteria, and actinomycetes that colonize the inner plant tissues without causing any pathological or harmful symptoms^7^. Recently, endophytic microbes have emerged in the field of nano-biosynthesis due to their efficient production of active metabolites potentially used for the manufacture of NPs of different shapes and sizes with great stability. In this context, endophytic fungi are superior to other fungal species in terms of the quantity and activity of the produced metabolites^8^. Previous studies indicated that endophytes could accomplish many biologically active secondary metabolites within the host plant such as flavonoids, alkaloids, saponins, sesquiterpenes, cyclopeptides, polyketones, organic acids, and lactones. Furthermore, the host plant and its endophytic symbionts share many biological properties such as anticancer, antimicrobial, anti-HIV, and anti-inflammatory activities^9^.

*Allium sativum* L. (Garlic) is a perennial plant extensively used for more than 4000 years as a curing agent in traditional medicine. The Egyptian papyri recorded recipes for the use of garlic in the treatment of snake bites, rhinitis, and heart disorders. In ancient Greece, garlic was used to treat lung and intestinal problems. It was also used in World War II to treat ulcers and wounds of the wounded. In general, garlic has many antifungals, antimicrobial, anticancer, antiprotozoal, antihypertensive, anticoagulant, anticonvulsant, anticoagulant, antipyretic, antipyretic, analgesic, and antioxidant properties^10^.

Different fungal species have been used as catalysts for the biosynthesis of Se-NPs including *Trichoderma harzianum*, *Aureobasidium pullulans*, *Phoma glomerata*, and *Mortierella humilis*, in addition to the endophytic fungi of *Aspergillus quadrilineatus*, *Aspergillus ochraceus*, *Aspergillus terreus*, and *Fusarium equiseti*^11,12^. Recently, the promising medical applications of myco-synthesized Se-NPs derived from *Penicillium citrinum* and their efficacy as anti-cancer and antioxidants have been reported^13^. Moreover, the biogenic Se-NPs obtained by the endophyte *Penicillium crustosum* demonstrated powerful anticancer and wide spectrum antimicrobial efficiency (against Gram-negative, Gram-positive bacteria, and four different *Candida* species) and durable catalytic activity for methylene blue degradation. These activities were more pronounced under light illumination than in dark conditions^14^. In addition, mosquitoes are considered the main vector for the transmission of causative agents for human and animal diseases such as viruses, protozoa, fungi, and bacteria. Deadly diseases such as yellow fever, dengue, filariasis, malaria, chikungunya, West Nile virus, and Zika virus are mosquito vector-borne diseases^15^. Se-NPs especially synthesized by green approaches, due to their low negative impacts on humans and the ecosystem, exhibit high mosquitocidal activity^16^.

Accordingly, the current study was designed to explore the ability of endophytic fungi to fabricate Se-NPs in an easy, efficient, and environmentally safe manner. Firstly, different endophytic fungal strains were isolated from garlic tissues and identified. Their potential in the biosynthesis of Se-NPs was explored. Next, the as-formed NPs were characterized using UV–Vis spectroscopy, Fourier transforms infrared (FT-IR), X-ray diffraction (XRD), Transmission Electron Microscopy (TEM), Dynamic light scattering (DLS), and zeta potential. Finally, their antibacterial, anti-*Candida*, antioxidant, anticancer, biocompatibility, and larvicidal properties were investigated.

## Results and discussion

### Isolation and primary identification of root endophytic fungi

**Figure 1:**
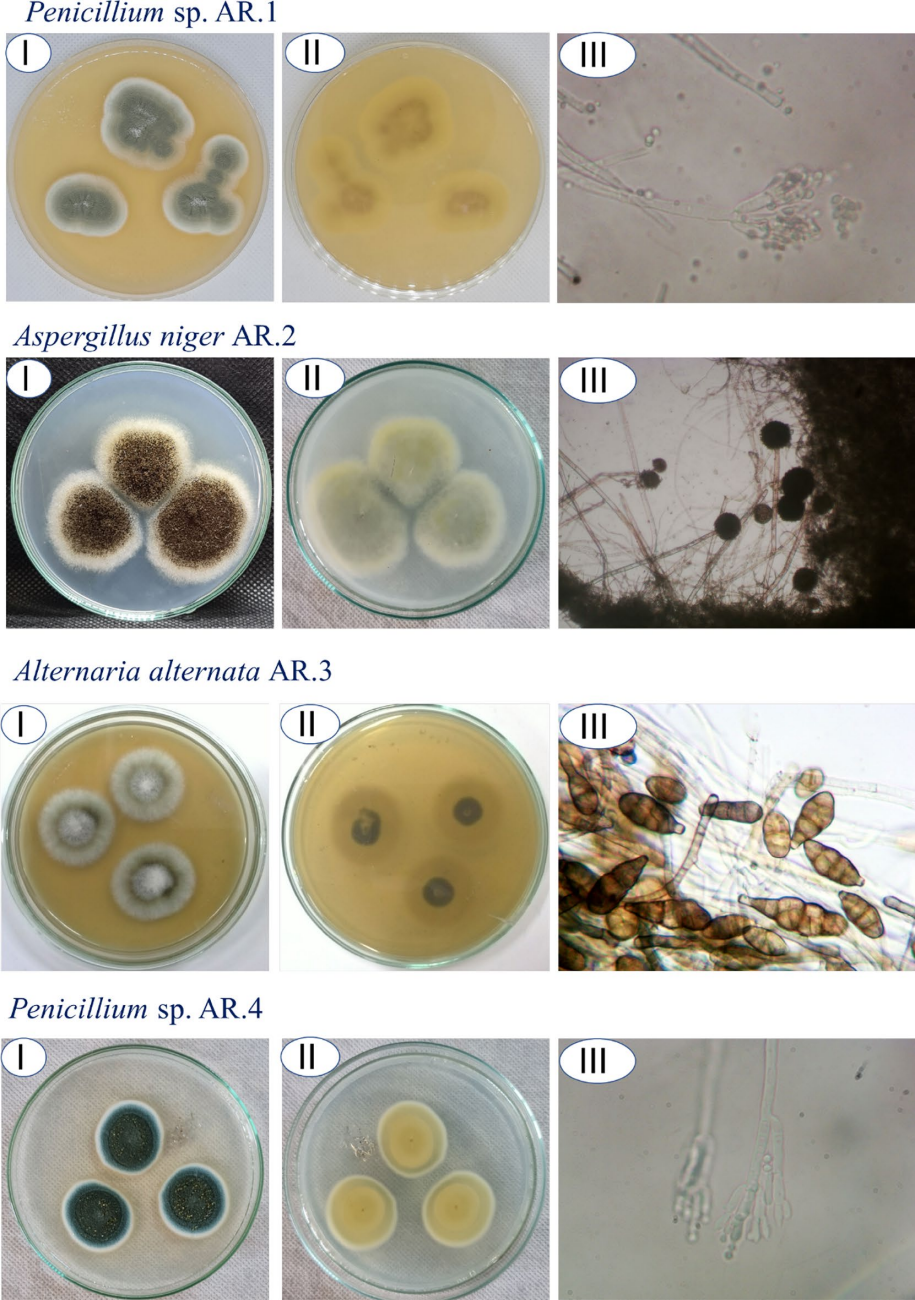
Isolation and primary identification of fungal strains isolated from the root of *Allium sativum.*

Endophytic fungi are one of the significant organisms’ having a wide range of biomedical and biotechnological applications including the production of active metabolites, utilization as biofertilizers due to their plant growth-promoting activity, phytopathogenic control, and green synthesis of nanomaterials of unique properties^17,18^. In the current study, the roots of garlic were used as the source for the isolation of endophytic fungal strains. Four fungal isolates designated as AR.1–AR.4 were obtained from collected healthy roots. These strains were identified using traditional methods based on cultural and microscopic characterizations as *Penicillium* sp. (AR.1), *Aspergillus niger* (AR.2), *Alternaria alternata* (AR.3), and *Penicillium* sp. (AR.4) (Fig. 1). In a similar study, twelve endophytic fungal strains belonging to *Aspergillus* spp., *Alternaria* spp., *Penicillium* spp., *Cladosporium* sp., *Chaetomium* sp., and *Fusarium* sp. were isolated from *Allium sativum*^19^.

### Screening for Se-NPs biosynthesis and identification of the most potent endophytic fungal strain using molecular analysis

Recently, researchers tended to produce new active compounds with an eco-friendly approach. Among these active compounds, nanomaterials possess high activity in various fields of agriculture, medicine, and industry^20^. The synthesis of these materials by green approaches is preferred to avoid the negative impacts originating from chemical and physical approaches^21^. Endophytic microbes including fungi, bacteria, and actinomycetes are considered promising sources for the green synthesis of nanomaterials due to the secretion of huge active metabolites that are used as reducing and capping agents^8^. In the current study, the efficacy of endophytic fungal strains in the synthesis of Se-NPs was investigated. Once adding metal precursor (Na_2_SO_3_) to the fungal biomass filtrate, the color was changed from colorless to red color which increased gradually, indicating the formation of Se^0^ due to the reduction of SeO_3_^2–^. The previous mixture remained for 24 h in dark conditions to confirm the complete reduction of metal and no further color change. Recently, the complete reduction of Na_2_SO_3_ by the action of metabolites secreted by the endophytic fungal strain, *P. crustosum,* whereas the Se^0^ form was completed after 24 h of incubation^14^. Also, the fabrication of Se-NPs through the reduction of Na_2_SO_3_ using metabolites secreted by *Trichoderma atroviride* was observed after 24 h, and no further color change was noticed^22^.

**Figure 2:**
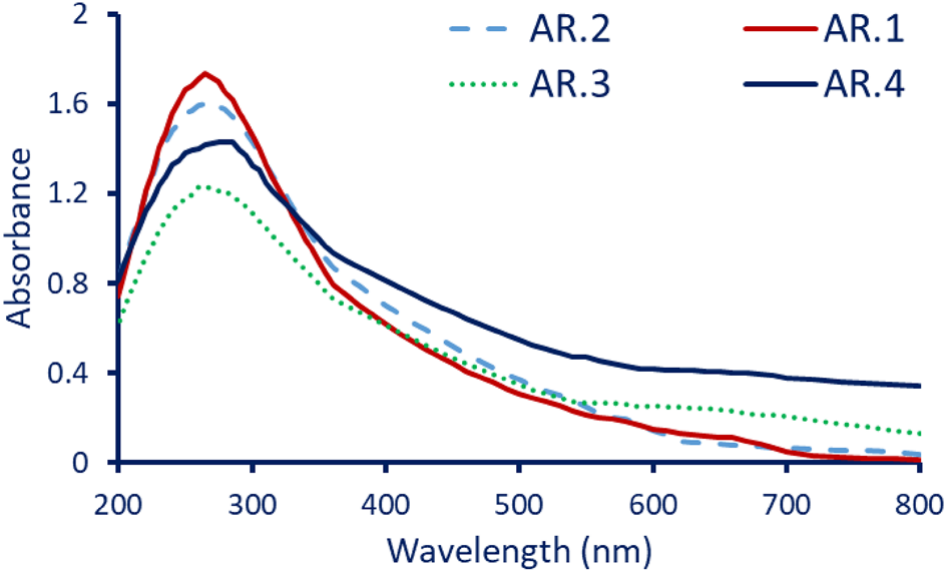
UV–Vis spectroscopy of Se-NPs fabricated by four endophytic fungal strains to select the most potent isolates based on maximum SPR.

Herein, after 24 h, the absorbance of the formed color was measured to detect the maximum surface plasmon resonance (SPR). As shown, the incubation period showed a positive impact on the color intensity without any shifting in the range of SPR. Figure 2 depicts that the absorption peak was recorded at wavelengths of 270 nm, 265 nm, 265 nm, and 280 nm for endophytic fungal strains of AR.1, AR.2, AR.3, and AR.4, respectively. Interestingly, the maximum color intensity and the maximum SPR absorption peak were recorded for strain AR.1. The obtained results were compatible with those reported that the maximum SPR for Se-NPs synthesized by fungal strains lies in the range of 250–300 nm. For instance, among 75 endophytic fungal strains, only four strains identified as *Aspergillus quadrilineatus, A. ochraceus, A. terreus*, and *Fusarium equiseti* showed high activity for Se-NPs synthesis based on color change and maximum SPR which appeared at 265 nm^11^. Also, the maximum SPR of Se-NPs fabricated by *Penicillium corylophilum* and endophytic fungal strain *P. crustosum* was observed at 275 and 270 nm, respectively^14,23^.

**Figure 3:**
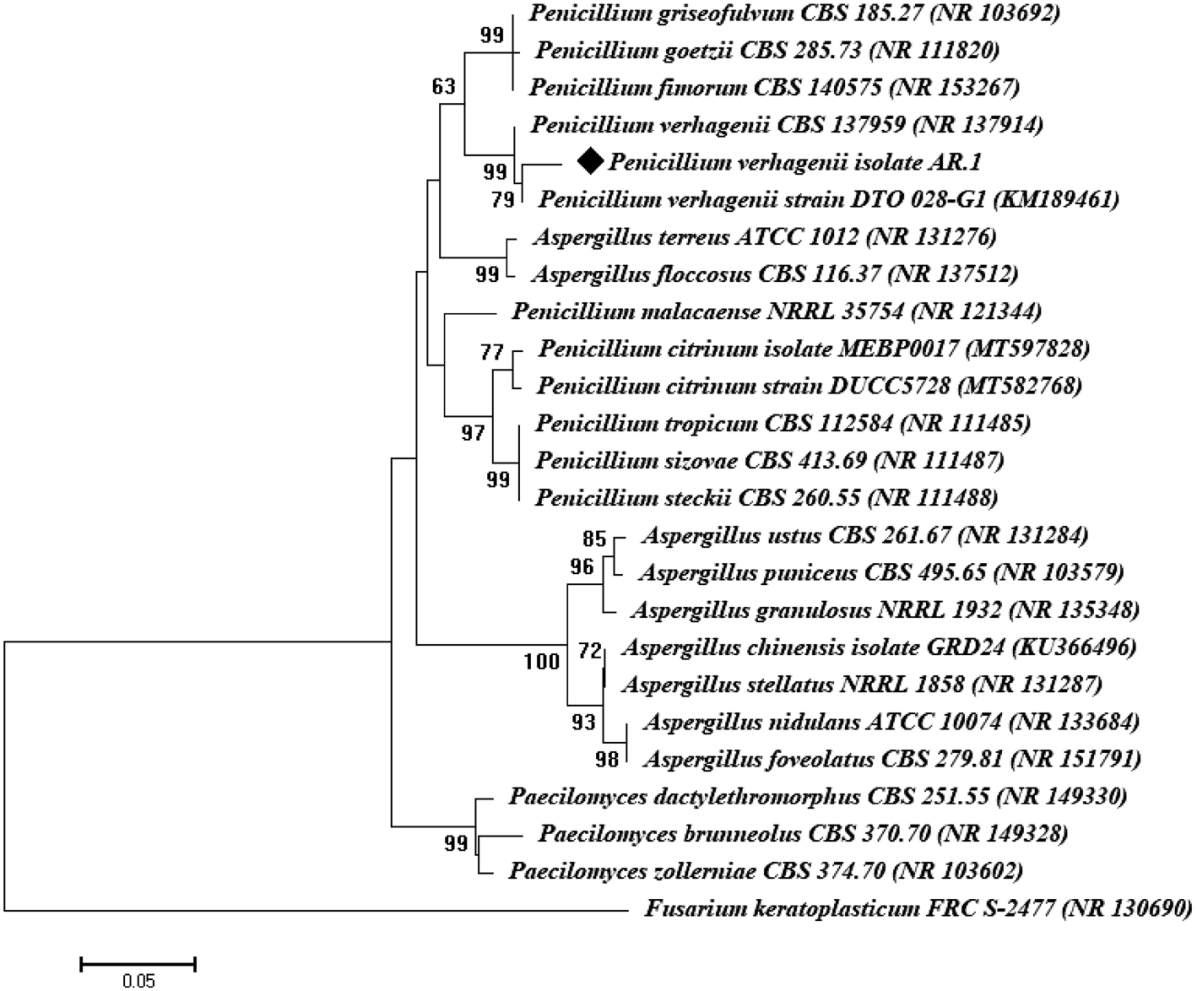
Phylogenetic tree of the most potent endophytic fungal strain.

According to the data of UV–Vis spectroscopy, the endophytic fungal strain designated as AR.1 was selected as the most potent strain for green synthesis of Se-NPs. This strain has undergone molecular identification based on amplification and sequencing of internal transcribed spacer (ITS) genes and was identified as *Penicillium verhagenii* (Fig. 3). The ITS sequence of the endophytic strain AR.1 was deposited in GenBank under the accession number of OP471232. *Penicillium* is composed of distinct species which are advantaged by their ability to produce various active metabolites that are used as reducing and stabilizing agents during green synthesis ^24^.

### Characterization of Se-NPs fabricated by *Penicillium verhagenii* strain AR.1

**Figure 4:**
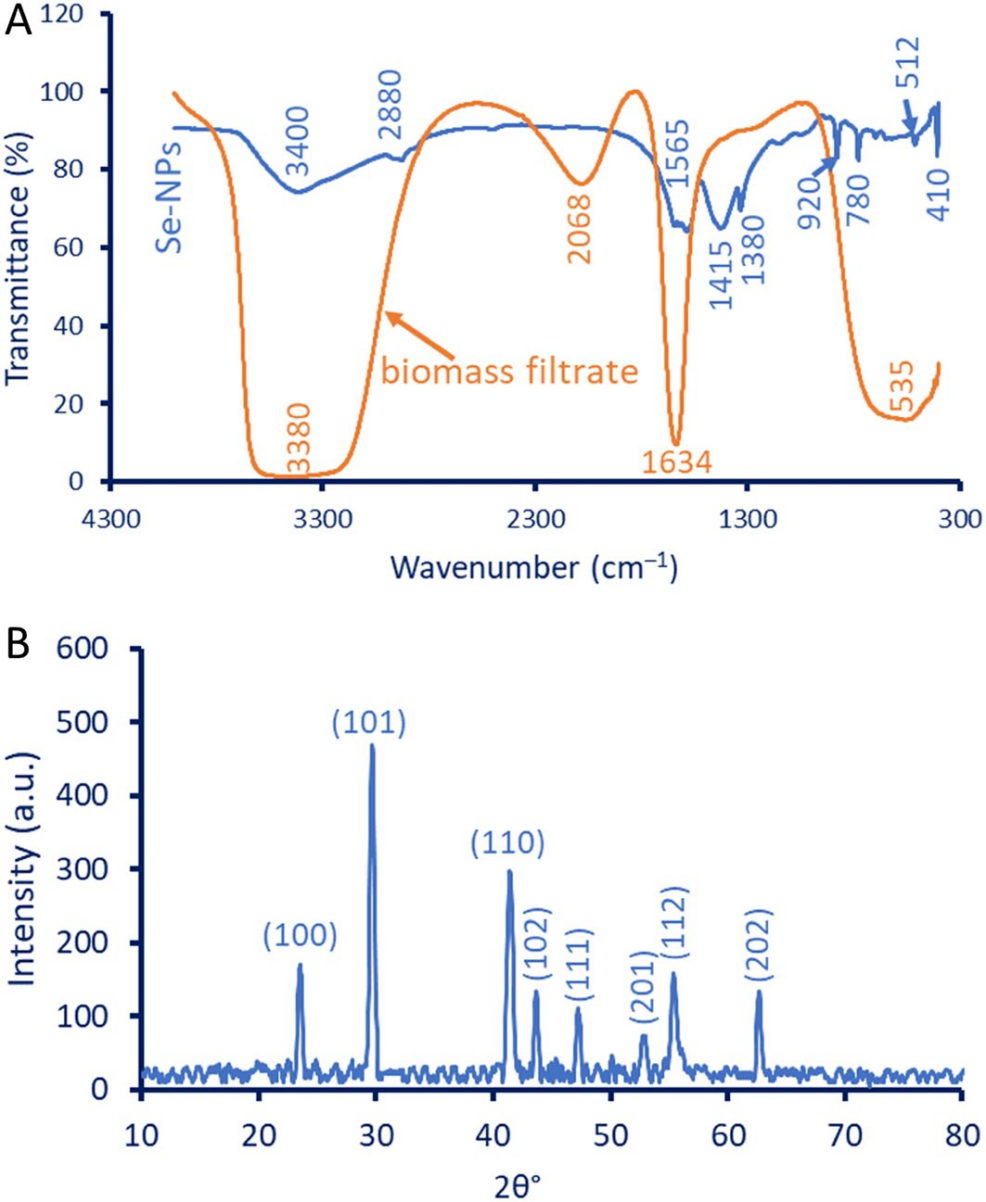
Characterization of synthesized Se-NPs using FT-IR (**A**) and XRD (**B**).

As mentioned, the color change followed by the detection of maximum SPR using UV–Vis spectroscopy was the first monitor for the successful formation of Se-NPs. The endophytic fungal isolate AR.1 showed the highest color intensity and absorption peak at 270 nm which corresponds to the SPR for Se-NPs. The functional groups exist in fungal biomass and their activity in reduction and stabilizing synthesized Se-NPs were investigated by Fourier transform infrared (FT-IR) analysis. As shown, the fungal biomass filtrate contained only four peaks at wavenumbers 3380, 2068, 1634, 535 cm^–1^, whereas in the case of Se-NPs, the number of peaks increased to nine at wavenumbers 3400, 2880, 1565, 1415, 1380, 920, 780, 512, and 410 cm^–1^ (Fig. 4A). The strong and broadness peak at 3380 cm^–1^ could be attributed to the O–H and N–H groups of proteins and amino acids^25,26^, this peak was shifted to 3400 cm^–1^ at Se-NPs. The broadness peak at 2068 cm^–1^ is related to the carbohydrate moiety secreted by endophytic fungal strains. Moreover, the peak at 1634 cm^–1^ corresponds to the carbonyl (C=O) group that is overlapped with the stretching NH group of polysaccharides present in biomass filtrate^4^. This peak was shifted to 1565 cm^–1^ after the green synthesis of Se-NPs. A peak at 535 cm^–1^ in biomass filtrate could be attributed to stretching C–l of the halo compound. The presence of other peaks in the FT-IR chart of Se-NPs could be related to the interaction between metabolites in biomass filtrate with sodium selenite during the reduction and capping of as-formed Se-NPs. The medium peak at 2880 cm^–1^ signifies C–H stretching alkane, whereas the medium peaks in the range of 1380–1420 cm^–1^ could have corresponded to the bending O–H of carboxylic acid^27,28^. The peaks in the range of 400–800 cm^–1^ are corresponding to bending and stretching Se-O which resulted from the reaction of Se-NPs with carbonyl groups ultimately forming a coating layer around the Se-NPs surface that prevents the aggregation and agglomeration as reported previously^29^. Based on FT–IR analysis, the presence of different metabolites in fungal biomass filtrates such as proteins, polysaccharides, carbohydrates, and amino acids exhibited a crucial role in the reduction of sodium selenite to form Se-NPs followed by forming a coating that enhances the NPs stability and prevents the aggregation.

The crystalline structure of as-formed Se-NPs was investigated by X-ray diffraction (XRD) analysis (Fig. 4B). As shown the XRD pattern displayed eight absorption peaks of (100), (101), (110), (102), (111), (201), (112), and (202) which matched Bragg diffraction at 2θ values of 23.5°, 29.3°, 41.3°, 45.51°, 52.53°, 55.71°, and 62.74°, respectively. The obtained XRD pattern was matched with those that confirmed the crystalline structure of Se-NPs according to JCPDS standard card No. 06-0362. The obtained XRD pattern is compatible with published studies on the green synthesis of Se-NPs^14,30,31^. The absence of additional peaks in the XRD chart indicated the high purity of the synthesized Se-NPs (verified by EDX spectra). The average crystallite size of Se-NPs was calculated based on XRD analysis using Debye–Scherrer’s equation to be 55 nm. In a recent study, the average crystallite sizes fabricated by four endophytic fungal stains, *Aspergillus quadrilineatus, A. ochraceus, A. terreus*, and *Fusarium equiseti* were calculated based on XRD analysis to be 55.4, 45.2, 30.9, and 30.1 nm, respectively^11^.

**Figure 5:**
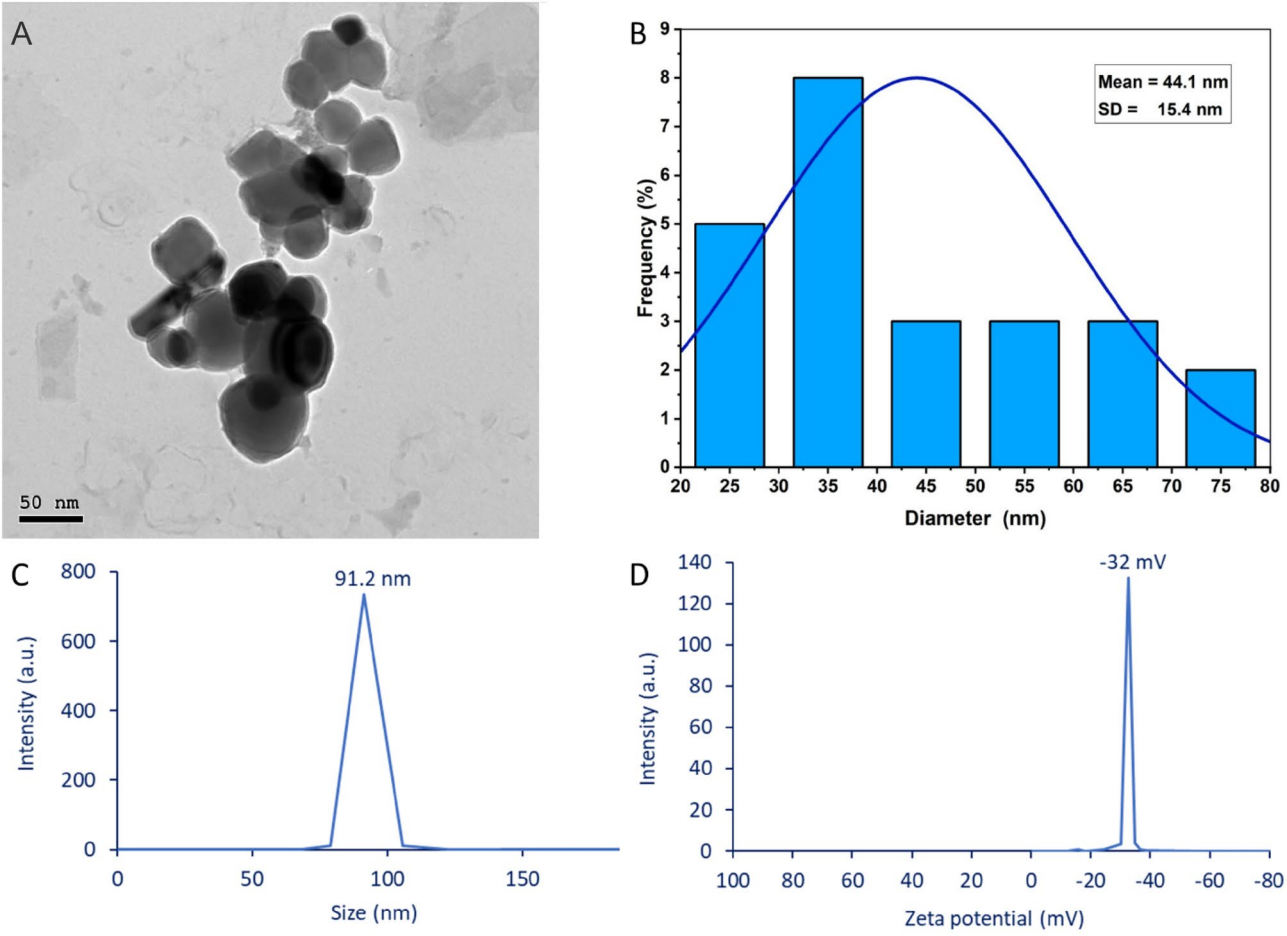
(**A**) Transmission Electron Microscopy showing the spherical shape, (**B**) size distribution, (**C**) the dynamic light scattering, and (**D**) Zeta potential analysis of Se-NPs fabricated by endophytic fungal strain *P. verhagenii.*

The morphological characteristics of fungal-mediate synthesized Se-NPs such as size, shape, and aggregation are principal factors that affect biological activities were investigated by transmission electron microscopy (TEM) analysis. Figures 5A and B displayed the spherical shape of synthesized Se-NPs that are well arranged without agglomeration and have sizes in the range of 25–75 nm with an average size of 44.1 ± 15.4 nm. The TEM image of Se-NPs formed at a concentration of 1.5 mM by harnessing metabolites of *Acinetobacter* sp. SW30 showed the successful fabrication of a spherical shape with a size of 78 nm^32^. The incorporation of NPs in different applications mainly depends on various factors such as capping agent, surface charge, shape, size, and agglomeration^21^. As size decreased, the activity increased. For instance, Se-NPs synthesized by biomass filtrate of *Pantoea agglomerans* showed higher antioxidant activity at smaller sizes^33^. Also, the activities of NPs are varied according to shape. For instance, Se-NPs showed high antioxidant activity for cubic shape and high antimicrobial activity for spheric shape^34^.

The size of fungal-based Se-NPs in colloidal solution was detected by dynamic light scattering (DLS). As shown, the average hydrodynamic size of synthesized Se-NPs was 91.2 nm (Fig. 5C). In the current study, the average size obtained by DLS is bigger than those obtained by TEM and XRD. This finding could be attributed to the DLS measuring hydrodynamic residue (hydrated state) whereas TEM calculates the diameter of the solid state^35^. Also, DLS is affected by coating agents and non-homogenous distribution which increases the sizes^36,37^. In a similar study, the average particle sizes of Se-NPs obtained by TEM were 15–40 nm whereas those obtained by DLS were 20–60 nm ^4^ which was explained by the hydrodynamic coating around the particles.

The stability of synthesized Se-NPs was investigated by Zeta potential which measures the electric charge on the NP's surface. In the current study, the fungal-based Se-NPs have a Zeta potential value of -32 mV which refers to the high stability (Fig. 5D). Similarly, the Zeta potential value of Se-NPs fabricated by aqueous extract of *Carica papaya* was −32 mV^38^. The stability of Se-NPs fabricated in the current study could be attributed to the presence of a negative charge which increases the negative electrostatic force between the particles and hence enhances the dispersion. Also, the stability of NPs can be classified according to Zeta potential values as follows: unstable, moderate, stable, and high stability for the values of ± 0–10, ± 10–20, ± 20–30, ˃ ± 30, respectively^39^. Moreover, the absence of double charges (positive and negative) and the presence of a single charge (negative) on the NPs surfaces could increase the stability as the dispersion between the particles bears the same charge to avoid aggregation. Dhanjal and Cameotra^40^ reported that the presence of positive charges on the surface of some particles and negative charges on others in the same solution enhances their aggregation.

### Antimicrobial activity

Most of the mortality or morbidity worldwide is caused due to microbial infectious diseases. Bacterial infections are increasing day-to-day due to the abuse of antibiotics leading to the emergence of antibiotic-resistant strains^6,41^. Further, *Candida* strains are opportunistic fungi and are considered the most common agent for infectious diseases such as oral candidiasis, candidemia, vaginitis, systematic infections, and cutaneous candidiasis in immunocompromised patients^42^. Therefore, it is important to construct new active compounds, safe and cost-effective to overcome these challenges. Selenium ions exhibited efficacy as antimicrobial agents and were previously used as additives to antidandruff shampoos due to their promising antifungal activity^43^. Unfortunately, they are used in little amounts due to toxicity to mammalian cells^38^. Therefore, the researchers focused on reducing their toxicity by converting them to nano-scale.

**Figure 6:**
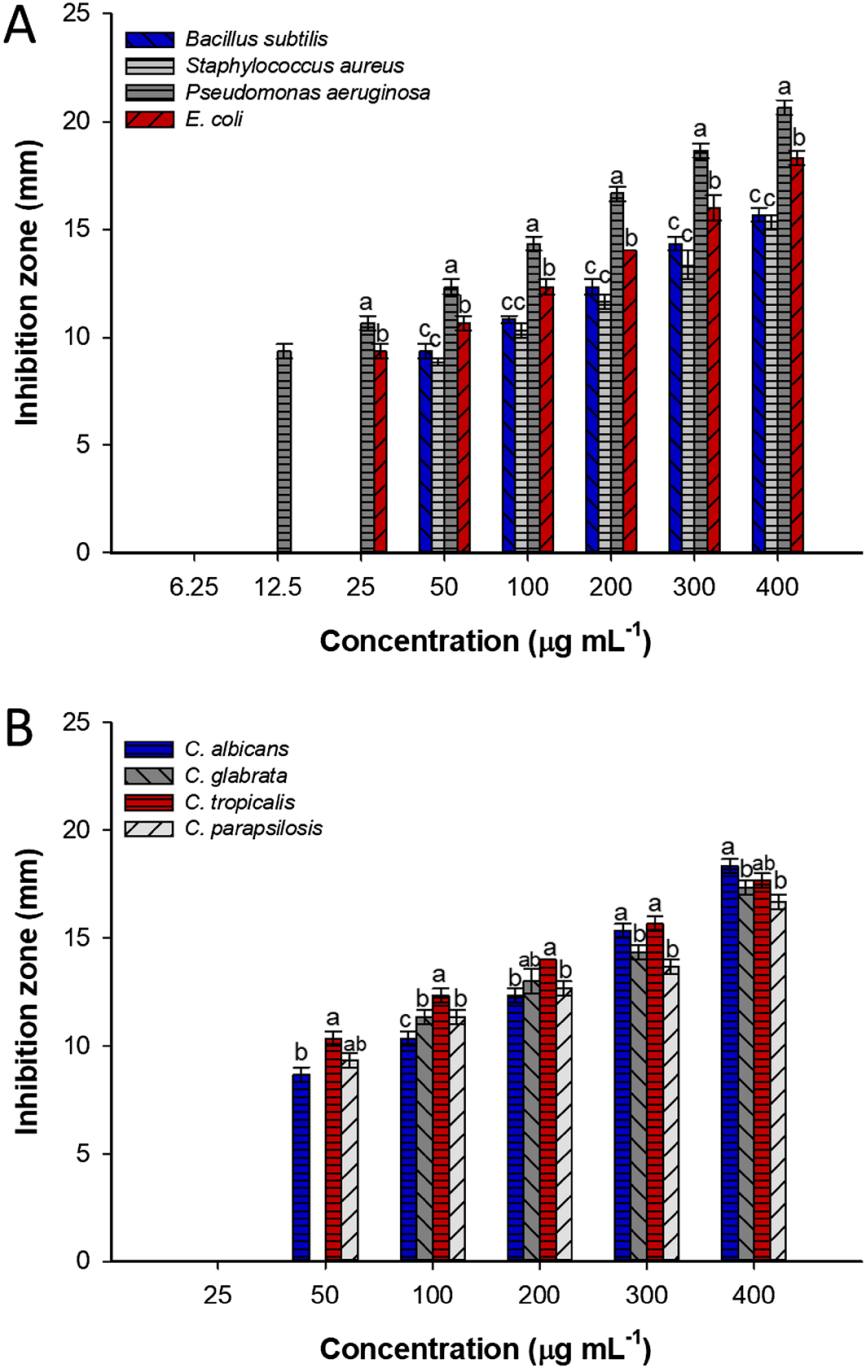
Antimicrobial activity of Se-NPs fabricated by an endophytic fungal strain of *P. verhagenii* against Gram-positive and Gram-negative bacteria (**A**) and unicellular fungi (**B**) at different concentrations. The different letters at the same concentration denotes the data are significant differences (*p* ≤ 0.005) (n = 3).

In the current study, the activity of green synthesized Se-NPs to inhibit the growth of pathogenic bacteria represented by *Escherichia coli*, *Pseudomonas aeruginosa*, *Bacillus subtilis*, and *Staphylococcus aureus* as well as different pathogenic *Candida* species designated as *C. albicans, C. glabrata, C. tropicalis,* and *C. parapsilosis* were investigated by well diffusion method. Data analysis showed that the antimicrobial activity of Se-NPs was concentration-dependent. The obtained findings were compatible with the published literatures. For instance, the Se-NPs formed by the action of the metabolites in the aqueous extract of *Ceropegia bulbosa* displayed high antimicrobial activity against *B. subtilis* and *E. coli* at 100 µg mL^–1^ followed by concentrations of 75, 50, and 25 µg mL^–1^^16^. In the current study, the highest antibacterial and anti-*Candida* activity was recorded at 400 µg mL^–1^ with inhibition zones of 15.7 ± 0.6, 15.3 ± 0.6, 20.7 ± 0.7, 18.3 ± 0.6, 18.3 ± 0.6, 17.7 ± 0.5, 17.3 ± 0.5, and 16.7 ± 0.6 mm for *B. subtilis*, *S. aureus, P. aeruginosa, E. coli, C. albicans, C. tropicalis, C. glabrata,* and *C. parapsilosism* respectively (Fig. 6A,B). The activity was decreased at low concentrations to 12.3 ± 0.6, 11.7 ± 0.5, 16.7 ± 0.7, 14.0 ± 0.0, 12.3 ± 0.6, 14.0 ± 0.0, 13.0 ± 1.0, and 12.7 ± 0.6 at 200 µg mL^–1^ of synthesized Se-NPs for the same sequence of above-mentioned test organisms. The as-formed Se-NPs by leave aqueous extract of *Withania somnifera* showed antibacterial activity against *B. subtilis*, *S. aureus*, and *Klebsiella pneumoniae* with the zone of inhibitions of 14.0 ± 0.0, 19.7 ± 0.6, and 12.0 ± 0.0 mm, respectively with no activity against *E. coli*^6^. Also, the Se-NPs fabricated by biomass filtrate of *Penicillium corylophilum* showed broad antibacterial activity against Gram-positive bacteria (*B. subtilis* and *S. aureus*) and Gram-negative bacteria (*P. aeruginosa* and *E. coli*)^23^.

The minimum inhibitory concentration (MIC) is the lowest concentration that has the efficacy to inhibit microbial growth. The choice of bioactive compounds to be incorporated into the biomedical sector is dependent on the evaluation of MIC value^44^. Herein, the synthesized Se-NPs showed varied MIC values based on the organism. For instance, the MIC value for Gram-positive bacteria was 50 µg mL^–1^, whereas it was 12.5 and 25 µg mL^–1^ for Gram-negative bacteria, *P. aeruginosa* and *E. coli,* respectively (Fig. 6A). On the other hand, the MIC value for tested unicellular fungi were in the ranges of 50–100 µg mL^–1^ (Fig. 6B). Recently, the MIC value of the green synthesized Se-NPs against *Candida* species was varied to be in the range of 25–200 µg mL^–1^ according to the type of specie^14^. Also, the antibacterial activity and MIC values were varied according to the biosynthetic approach. For instance, the MIC value of Se-NPs synthesized by *Aspergillus quadrilineatus* and *A. ochraceus* against *E. coli* was 62.5 µg mL^–1^, whereas it was 250 µg mL^–1^ for those fabricated by *A. terreus* and *Fusarium equiseti*^11^. This finding can be attributed to the capping agent secreted by microorganisms or plants that have a crucial role in stabilizing the nanomaterials^8,45^. Based on the obtained data, the as-formed Se-NPs by endophytic fungal strain *P. verhagenii* displayed high antimicrobial activity toward pathogenic bacteria and *Candida* spp.

The antibacterial activity of Se-NPs could be related to the cell wall structure, which in Gram-positive strains is composed of thick layers of peptidoglycan compared to thin layers in Gram-negative strains. This difference can affect the diffusion of NPs inside the cells. The thick peptidoglycan layer can prevent or delay the diffusion of Se-NPs leading to less antimicrobial activity toward Gram-positive bacteria than Gram-negative bacteria^46^. The activity toward Gram-positive bacteria can be attributed to the high electrostatic repulsion of Se-NPs toward the negative charge of lipopolysaccharide that exists in high amounts in Gram-negative bacteria than Gram-positive. This lead to high deposition of Se-NPs on the surface of Gram-positive strains that ultimate in cell death^47^. Another antimicrobial mechanism of Se-NPs could be the production of reactive oxygen species (ROS) upon entry into microbial cells. These ROS such as H_2_O_2_, O_2_^•–^, and ^•^OH can destroy the selective permeability function of the cytoplasmic membrane, increase the stress inside the cell, lead to inhibit DNA replication, destroy protein synthesis, and inhibit normal cell metabolism. All these dysfunctions lead to cell death^48,49^.

Interestingly, the fungal-mediated green synthesis of Se-NPs exhibited high activity against different strains of *Candida*. This finding could be related to its activity to destroy the sterol profile in the *Candida* cell wall by inhibiting the ergosterols biosynthesis pathway^50^. Also, a high accumulation of Se-NPs onto the *Candida* cell wall leads to the reaction of Se with sulfur-containing amino acids such as methionine and cysteine^51^. As a consequence of this interaction, the new structure which was S-Se-S was formed and modified the structure of proteins leading to the blocking of its catalytic functions^52^.

### Antioxidant activity

**Figure 7:**
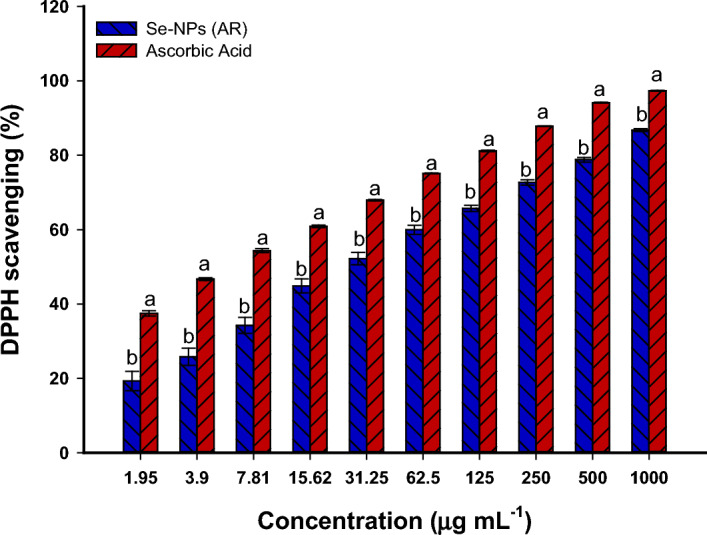
DPPH-scavenging activity of Se-NPs synthesized by endophytic *P. verhagenii* compared to ascorbic acid as control.

The scavenging activity of free radicals by green synthesized Se-NPs was investigated by the DPPH method compared to the control (ascorbic acid) (Fig. 7). Data analysis displayed that the free radicals scavenging activity is a concentration-dependent nature. The activity is directly proportioned with green synthesized Se-NPs concentration. The obtained finding was compatible with published literatures that reported the scavenging activity of Se-NPs fabricated by plants, fungi, and actinomycetes was dependent on their concentrations^4,11,53^. The maximum DPPH-scavenging activity was recorded at 1000 µg mL^–1^ with percentages of 86.8 ± 0.6% compared to ascorbic acid at the same concentration which recorded a percentage of 97.3 ± 0.2% (Fig. 7). The DPPH-scavenging activity reached 19.3 ± 4.5% at the lowest Se-NPs concentration of 1.95 µg mL^–1^. This indicates the synthesized Se-NPs possess antioxidant activity at low concentrations. In a similar study, Se-NPs fabricated by endophytic fungal strains of *A. quadrilineatus, A. ochraceus, A. terreus,* and *F. equiseti* exhibited DPPH-scavenging activity with percentages of 93.8 ± 9.5, 83.6 ± 6.3, 79.2 ± 9.3, and 79.8 ± 4.7%, respectively at 1000 µg mL^–1^ compared to 100% scavenging activity for ascorbic acid. These percentages reached 25.8 ± 2.1, 28.4 ± 2.6, 18.0 ± 3.5, and 10.3 ± 2.1% at a concentration of 25 µg mL^–1^ compared to the control (21.3 ± 1.5%) at the same concentration^11^. In the current study, the values of EC_50_ (effective concentration for scavenging 50% of free radicals) were 28.7 ± 1.6 µg mL^–1^ and 5.4 ± 0.8 µg mL^–1^ for Se-NPs and ascorbic acid, respectively. Our data was incompatible with those recorded that the EC_50_ of Se-NPs synthesized by biomass filtrate of fungal strain *Monascus purpureus* was 85.9 µg mL^–1^^54^, indicating the promising antioxidant activity of Se-NPs synthesized herein.

Antioxidant substances or free radical scavengers are those that prohibit cell damage caused by unstable molecules or free radicals synthesized under stresses such as pathogens, contaminants, radioactive substances, toxins, etc.^55^. The main symptoms of these free radicals are rheumatism, immune dysfunction, Parkinson’s, leukemia, heart attack, metabolic disorders, and respiratory failure^56^. The origin of these free radical scavengers is either natural sources such as phenolics, flavonoids, phytoestrogens, and tannins, or synthetic sources such as nanomaterials^38^. Metal and metal-oxide nanoparticles are characterized by their ability as free radical scavengers^33,56^. The activity of Se-NPs as antioxidants could be attributed to their efficacy in the upturn of selenoenzymes such as glutathione peroxidase which protect the cells from the deleterious effect of free radicals under *in-vivo* conditions^4,45^. Also, the antioxidants of NPs could be due to the inhibition and neutralization of the DPPH free radicals' formations by electron transfer^57^. Moreover, the unique properties of NPs especially high surface-to size can enhance antioxidant activity^58^.

### Anticancer and biocompatibility

**Figure 8:**
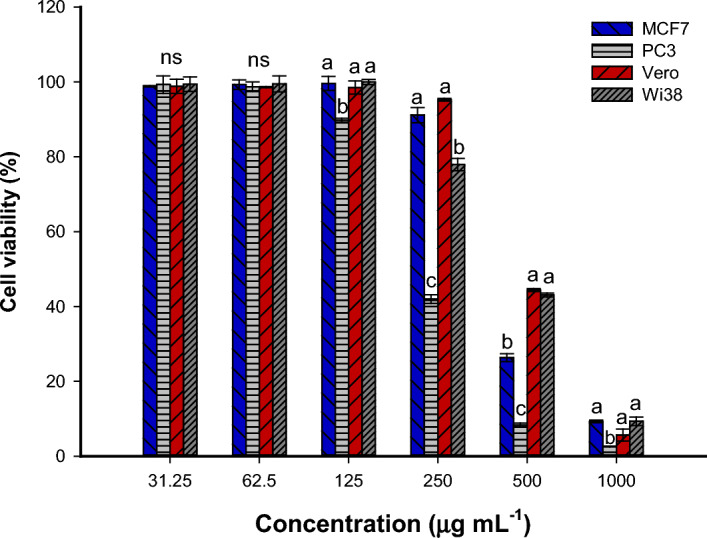
Cell viability using the MTT assay method of cancer cells (PC3 and MCF7) and normal cells (Vero and WI38) after treatment with various concentrations of Se-NPs.

The anticancer activity of green synthesized Se-NPs was investigated against two cancer cells designated as MCF7 and PC3, whereas the biocompatibility was assessed toward two normal cells represented as Vero and WI38. The cell viability and cellular proliferation due to Se-NPs treatment were assessed by the MTT assay method. Data analysis showed that the endophytic fungal strain-mediated green synthesis of Se-NPs has a promising anticancer activity against tested cancer cell lines in a concentration-based manner. At low concentrations (≤ 62.5 µg mL^–1^), the Se-NPs have no significant effects on the viability of cancer and normal cell lines (the cell viability in the range of 89–99% for all cells), whereas, at a concentration of 125 µg mL^–1^, the viability of PC3 was 89.7 ± 0.9% compared to cancer cell lines of MCF7 (99.6 ± 3.2%) and two normal cell lines (98.4 ± 3.1% and 99.9 ± 1.2% for Vero and WI38, respectively) (Fig. 8). By increasing the Se-NPs concentration to 500 µg mL^–1^, the viability was highly decreased for cancerous cell lines that reached 26.3 ± 1.8% and 8.3 ± 0.9% for MCF7 and PC3, respectively compared to the viability of normal cell lines (44.4 ± 0.7% and 43.1 ± 0.9% for Vero and WI38, respectively). Compatible with the obtained results, the activity of Phyto-synthesized Se-NPs was dependent on the concentrations against cancer cell lines of MCF-7, Caco-2, IMR-32, and normal cell line Vero^38^. Also, the antiproliferative activity of Se-NPs synthesized by harnessing metabolites of *Acinetobacter* sp. SW30 against 4T1, MCF7, NIH/3T3, and HEK293 were concentration-dependent manner^32^. Data analysis showed that the toxicity of fungal-mediated green synthesis of Se-NPs was the highest against PC3 compared to MCF7 (Fig. 8). On the other hand, the sensitivity of two normal cells toward various concentrations of Se-NPs was similar, except at 250 µg mL^–1^, the viability of WI38 was decreased compared to Vero cells.

The IC_50_ (concentration for inhibiting 50% of cellular viability) of Se-NPs was evaluated to be 225.7 ± 3.6, 283.8 ± 7.5, 454.8 ± 29.9, and 472.8 ± 5.8 µg mL^–1^ for PC3, MCF7, WI38, and Vero cell lines, respectively. The obtained data reveal the target orientation of Se-NPs toward cancerous cell lines at low concentrations compared to normal cell lines. This finding confirmed the ability to integrate Se-NPs in biomedical sectors at a concentration below 300 µg mL^–1^ to be more active against cancer cells with negligible effects on normal cells. The minimum cytotoxic effects of synthesized Se-NPs toward normal cells could be related to the normal redox balance as reported previously^59^.

The microscopic examination of cells treated with Se-NPs showed complete or partial destruction of a monolayer of epithelial cells under high concentrations (See supplementary data, Fig. S1, S2, S3, S4). Moreover, at these concentrations, the cells tend to be rounding or grainy, shrinking, buoyancy, and decrease in the total number. These negative impacts were reduced at low concentrations, especially for normal cells. The small size is the reason for cytotoxic activity due to their efficacy to penetrate the mammalian cell membranes and interact with cell components such as proteins, nucleic acids, and amino acids leading to cellular dysfunction^60^. Moreover, the presence of Se-NPs inside the cells can increase the generation of ROS which have harmful effects on mitochondria, and ultimately to apoptotic death^14^. Shiny et al., reported that the exposure of lung carcinoma cells (A549) to silver and platinum nanoparticles leads to destroying of the cellular cytoskeleton and hence the release of specific enzymes called cytosolic LDH enzymes which are responsible for the lysis of the cells^60^.

### Larvicidal activity

The use of chemical substances to control mosquito vectors had negative impacts not only on human health but also on the environment and the emergence of new resistant vectors^61^. Therefore, it is urgent to construct safe, eco-friendly, cost-effective, and rapid effect active compounds. Herein, fungal-based Se-NPs showed high mortality against the different instar larvae (I, II, III, and IV) of *Aedes albopictus* at various concentrations (10, 20, 30, 40, and 50 µg mL^–1^) (Table 1). As shown, the activity of Se-NPs against instar larvae was in a concentration-dependent manner. The obtained finding was compatible with literatures about the effect of green synthesized nanomaterials against mosquito vectors^62,63^. Data analysis showed that the highest mortality (85.1 ± 3.1%) for the I instar larva was recorded at a concentration of 50 µg mL^–1^, whereas this percentage was decreased by decreasing the concentration to reach 59.0 ± 2.0% at 10 µg mL^–1^. On the other hand, the as-formed Se-NPs showed high activity against IV instar larva with mortality percentages of 31.3 ± 1.5, 34.1 ± 0.0, 42.2 ± 1.0, 46.2 ± 1.6, and 51.0 ± 1.0% for concentrations of 10–50 µg mL^–1^, respectively.

**Table 1:** Larvicidal activity of Se-NPs at different concentrations against instar larvae of *Aedes albopictus.*

Larval stage	Concentrations µg mL^–1^	Percentage mortality	LC_50_ (LCL–UCL)	LC_90_ (LCL–UCL)	*x*^*2*^
I instar larva	10	59.0 ± 2.0	15.2 (7.1–26.6)	132.8 (110.1–201.9)	7.73
	20	63.1 ± 0.5			
	30	75.1 ± 1.0			
	40	81.2 ± 0.0			
	50	85.1 ± 3.1			
II instar larva	10	42.2 ± 1.1	33.6 (10.643–68.998)	138.9 (78.5–140.2)	8.54
	20	48.0 ± 1.0			
	30	54.2 ± 1.2			
	40	57.1 ± 1.0			
	50	67.2 ± 1.2			
III instar larva	10	35.21 ± 1.5	45.5 (13.3–46.2)	142.4 (150.6–245.4)	9.42
	20	41.53 ± 2.3			
	30	46.24 ± 1.0			
	40	56.00 ± 0.0			
	50	62.10 ± 1.4			
IV instar larva	10	31.3 ± 1.5	54.3 (14.2–55.5)	180.3 (180.6–265.4)	10.43
	20	34.1 ± 0.0			
	30	42.2 ± 1.0			
	40	46.2 ± 1.6			
	50	51.0 ± 1.0			

LC_50_ (concentration for 50% mortality); LC_90_ (concentration for 90% mortality); LCL (lower confidence limit); UCL (upper confidence limit); *x*^*2*^ (chi-square value). Data are represented by the means of five replicates ± SE.

Analysis of variance revealed that the LC_50_ and LC_90_ for I, II, III, and IV instar larva of *A. albopictus* were (15.2, 33.6, 45.5, and 54.3 µg mL^–1^) and (132.8, 138.9, 142.4, and 180.3 µg mL^–1^), respectively (Table 1). In a similar study, the LC_50_ and LC_90_ of plant-based Se-NPs against instar larva of *A. albopictus* were in the ranges of (15.2–52.3 mg L^–1^) and (132.7–178.3 mg L^–1^), respectively^16^. Se-NPs fabricated by leaf aqueous extract of *Clausena dentata* exhibited larvicidal activity toward *Culex quinquefasciatus, Aedes Aegypti*, and *Anopheles stephensi* with LC_50_ values of 99.6, 104.1, and 240.7 mg L^–1^, respectively^64^.

The activity of Se-NPs toward *A. albopictus* could be attributed to their efficacy to penetrate the cell membrane and interact with different cellular components leading to dysfunction^16^. Also, the presence of Se-NPs inside the cell can increase the oxidative stress that is ultimate in cell death by generating toxic ROS. Moreover, Se-NPs can destroy the cells by reacting with the -SH group of amino acids or phosphorus-containing nucleic acids^65^.

## Material and method

### Plant sample

The roots of garlic (*Allium sativum* L.) from healthy plants were collected and used as a source for the isolation of fungal endophytes. Our work complies with institutional, national, and international guidelines and legislation. Garlic is common worldwide and does not need permission or licenses as the species we are working with is a cosmopolitan crop that is not at risk or endemic according to IUCN. The root samples were collected from agricultural land in El-Menofia governorate, Egypt (30° 38′ 40.9″ N 30° 56′ 49.9″ E) under permission (number: EM2/2022) from the local agricultural office in the governorate. Roots of healthy garlic plants were collected and kept in sterilized polyethylene bags before being transferred to the Laboratory using an icebox. During sample collection, five individual plants were obtained, and from each plant, three individual roots were collected.

### Isolation of fungal endophytes

The collected roots are rinsed thrice with tap water to remove any adhering particles followed by rinsing with sterilized distilled H_2_O. After that, the roots were subjected to surface sterilization by immersing them into the following solutions in order: sterilized distilled H_2_O for 60 s, ethanol (70%) for 30 s, sodium hypochlorite (2.5%) for four minutes, ethanol (70%) for 30 s, and finally washing the roots with sterilized dis. H_2_O. To confirm the surface sterilization process, the sterilized dis. H_2_O from the last washing was inoculated into appropriate agar media for the growth of different microorganisms (nutrient agar for bacteria, Czapek Dox for fungi, and starch nitrate for actinomycetes). The inoculated plates were incubated at appropriate conditions and observed daily to check the growth of microbes. The absence of microbial growth in inoculated agar media indicates the success of the surface sterilization process.

The sterilized roots were cut into small segments (4 mm/segment) and ten parts per individual plant were put on the surface of the Czapek Dox agar plate (5 segments/plate) supplemented with chloramphenicol to suppress the bacterial growth. The plates were incubated at 25 ± 2 °C for 15 days and observed daily to check the appearance of fungal growth from internal plant tissues, hence picked up and re-inoculated into a new plate. The purified fungal isolates were identified based on morphological and cultural characteristics according to standard keys for *Penicillium* spp.^66^, *Aspergillus* spp.^67^, and *Alternaria* spp.^68^.

### Checking for the activity of isolated endophytic fungi to the biosynthesis of Se-NPs

The efficacy of isolated endophytic fungal strains to act as a biocatalyst to fabricate Se-NPs was investigated. One disk (10 mm) from each fungal strain was inoculated separately in 100 mL of Czapek Dox broth media and incubated at 25 ± 2 °C for 7 days under shaking conditions (150 rpm). At the end of the incubation period, the fungal biomass was collected by filtration of inoculated broth media using Whatman No.1 and washed thrice with sterilized dis. H_2_O to remove any adhering media components. Approximately 7 g of biomass collected for each fungal strain was suspended into 100 mL dis. H_2_O and incubated at 25 ± 2 °C for 24 h followed by centrifugation to collect the biomass filtrate which was used to fabricate Se-NPs as follows: 85.5 mg of metal precursor (Na_2_SO_3_) was dissolved in 10 mL dis. H_2_O and added to 90 mL fungal biomass to get a final concentration of 5 mM. The mixture was subjected to stirring at 40 °C for one hour and adjusted the pH at 8 using 1N NaOH, before being left at room temperature overnight under dark conditions. The conversion of color from colorless to ruby red color indicates the formation of Se-NPs followed by measuring their absorbance at a wavelength in the range of 200–700 nm to detect the maximum surface plasmon resonance (SPR)^31^. The fungal biomass filtrate without metal precursor was used as a control. The most potent fungal strain was selected due to its efficacy to form the highest ruby red color and maximum SPR value. The resultant was collected and rinsed thrice with dis. H_2_O before being undergone oven-dry at 200 °C for 4 h.

### Molecular identification of the most potent fungal isolate

The selected most potent endophytic fungal isolate designated as AR.1 was subjected to molecular identification through amplification and sequencing of ITS genes according to the protocol of White et al*.*^69^. The ITS gene was amplified using a primer of ITS1 (5′ CTTGGTCATTTAGAGGAAGTAA-3′) and ITS4 (5′ TCCTCCGCTTATTGATATGC 3′). The PCR mixture containing the following: 0.5 mM MgCl_2_, PCR buffer, 2.5 U Taq polymerase (QIAGEN, GERMANTOWN, MD-20874, USA), 0.5 µM of each primer, 2.25 mM dNTP, extracted genomic DNA (5 ng). The run was achieved using DNA Engine Thermal Cycler (PTC-200, BIO-RAD, USA) and adjusted at 94 °C for three minutes followed by 30 cycles at 94 °C for a half minute, 55 °C for a half minute, 72 °C for one minute, and finally 72 °C for ten minutes. The PCR products were checked using agarose gel (1%) before being sequenced on GATC Biotech [A company using a DNA sequencer (ABI-3730xl) as a partner of Sigma Aldrich, Cairo, Egypt]. The obtained sequences were compared by the database deposited in GenBank by ClustalX 1.8 software package (http://www.clustal.org/clustal2)^70,71^. The Phylogenetic analysis was achieved using the neighbor-joining method (MEGA v6.1) software, with confidence tested by bootstrap analysis (1000 repeats).

### Characterization of Se-NPs

Fourier transform infrared (FT-IR) (Cary-660 model) was used to detect the different functional groups in fungal biomass as well as in as-formed Se-NPs. In this analysis, 10 mg of synthesized Se-NPs was mixed with KBr and pressed to form a disk subjected to scanning at a wavelength in the ranges of 400–4000 cm^-1^^22^. The crystallographic structure of fungal mediated green synthesis of Se-NPs was assessed by X-ray diffraction (XRD, PANalytical-X’Pert-Pro-MRD) containing CuKα electrode as a source of X-ray (λ = 1.54 Å). The analysis was achieved at current and voltage of 30 mA and 40 kV respectively in the range of 2θ values of 10°–80°. The crystallite size was calculated based on XRD analysis by Debye–Scherrer’s equation as follows:

1 $$ \text{Average} \, \text{crystallite} \, \text{size} = \frac{{\text{K}\lambda}}{{\beta\cos \theta }} $$

where K is a Scherrer constant which was 0.94, λ is the wavelength of the X-ray which was 1.54, β is the full width of the diffraction peak at a half maximum, and θ is the diffraction angle.

The morphological characteristics (shape and size) were checked by transmission electron microscopy (TEM, JEOL, Ltd-1010, Tokyo, Japan). The green synthesized Se-NPs powder was suspended in H_2_O under ultrasonication and added a few drops on the TEM-carbon grid. The loaded grid was left to dry before being subjected to analysis^72,73^.

Dynamic light scattering (DLS) (Nano-ZS, Malvern Ltd, Malvern, UK) was used to investigate the size distribution in the colloidal solution. The synthesized Se-NPs were dispersed in a high pure solvent (MiliQ H_2_O) to prevent the appearance of shadow on the signal during scattering analysis. Moreover, the surface charge of synthesized Se-NPs was assessed using Zeta-sizer apparatus (Nano-ZS, Malvern, UK)^74^.

### Antimicrobial activity

The activity of fungal-based Se-NPs as antimicrobial agents was investigated toward a group of pathogenic microbes including *Escherichia coli* ATCC8739, *Pseudomonas aeruginosa* ATCC9027 (Gram-negative bacteria), *Bacillus subtilis* ATCC6633, and *Staphylococcus aureus* ATCC6538 (Gram-positive bacteria). Also, the activity was assessed against a group of clinical *Candida* strains designated as *C. albicans, C. glabrata, C. tropicalis,* and *C. parapsilosis* which were collected from Microbiology Laboratory, National Research Centre, Giza Egypt. The antimicrobial activity was assessed using the agar well diffusion method^75^. In this method, the selected bacterial and fungal strains were subculture on nutrient agar (Ready-prepared, Oxoid) and sabouraud dextrose agar plates (containing gL^-1^: dextrose, 40; peptone, 10; agar, 15), respectively for overnight at 35 ± 2 °C. After that, a single colony from each organism was picked up and spread uniformly by sterilized swab on the surface of the Muller-Hinton agar plate (Ready prepared, Oxoid) followed by making four wells (0.6 mm in diameter) in each plate. These wells were filled with 100 µL of the prepared Se-NPs solution (400, 300, 200, 100, 50, 25, 12.5, and 6.25 µg mL^–1^). The solvent system (DMSO) was running with the experiment as control. The filled plates were incubated at 35 ± 2 °C for 24 h. At the end of incubation time, the results were recorded as a diameter of a clear zone formed around each well. The lowest Se-NPs concentration that inhibits microbial growth (MIC value) was determined. The experiment was conducted in triplicate.

### Antioxidant activity

The antioxidant activity of fungal-mediated biosynthesis of Se-NPs was assessed by DPPH (2,2-diphenyl-1-picrylhydrazyl) method. In this method, various concentrations of biosynthesized Se-NPs (1.95–1000 µg mL^–1^) were prepared in high pure water (Milli-Q H_2_O). After that, one mL of prepared solution was added to a test tube containing one mL of DPPH (prepared in methanol) and 450 µL of Tris–HCl buffer (pH 7.4, 50 mM), mixed well before being incubated at 37 °C for a half-hour under shaking condition (100 rpm) in dark. Another set of experiments using ascorbic acid (positive control) was conducted under the same conditions /concentrations. Also, the negative control which was DPPH and Tris–HCl buffer in the absence of Se-NPs or ascorbic acid was running with the experiment under the same incubation conditions. At the end of the incubation period, the absorbance of the formed color was measured at 517 nm. The free radical scavenging percentages were calculated using the following Eqs.^6^:

2 $$ {\text{DPPH scavenging activity (\% ) }} = \, \frac{{{\text{Ab}}_{{\text{C}}} - {\text{Ab}}_{{\text{T}}} \, }}{{{\text{Ab}}_{{\text{C}}} }} \times {100} $$

where Ab_C_ and Ab_T_ are the absorbances of the control (ascorbic acid) and treatment (Se-NPs), respectively.

### Anticancer and biocompatibility test

#### A- Source of cell lines

The two cancer cells, designated as MCF7 (human breast cancer) and PC3 (prostate cancer cell), and two normal cells represented by Vero (monkey kidney epithelial cell) and WI38 (Human lung fibroblast) were purchased from the Holding Company for Biological Products and Vaccines (VACSERA), Cairo, Egypt.

#### B- MTT assay

The anticancer activity of Se-NPs against two cancer cells (MCF and PC3) and the biocompatibility test toward two normal cells (Vero and WI38) was assessed using the MTT assay method. In this method, each cell type was inoculated in 96-well tissue culture plates with intensity 1 × 10^5^ cells/100 µL/well and incubated at 37 °C for 24 h in a 5% CO_2_ incubator. Once the monolayer sheet was formed, it was rinsed twice with washing media and adding 100 µL of RPMI maintenance media with 2% serum. After that, the growing cells were treated with double-fold concentrations of Se-NPs (31.25–1000 μg mL^–1^) and incubated for 48 h. Three wells without Se-NPs were used as control. After the incubation period, the remaining media in each well was discarded and received 50 µL of MTT solution (5 mg mL^–1^ of phosphate buffer saline solution) and shaken thoroughly for 5 min before being incubated for 4 h at 37 °C. After the complete incubation period, the MTT solution was discarded, and adding 100 µL of DMSO (10%) was to dissolve the formed formazan crystal through shaking for 30 min. The absorbance of the formed color was measured at 570 nm by an ELIZA reader^76^. The morphological changes in the cells due to Se-NPs treatment were observed using inverted microscopy (Nikon, ECLIPSE Ts2, Shinjuku, Tokyo, Japan), whereas the cell viability percentages were calculated by the following equation:

3 $$ \text{Cell}\, \text{viability}\,(\% ) = \frac{{\text{Ab}_{\text{T}} }}{{\text{Ab}_{\text{C}} }} \times 100 $$

where Ab_T_ and Ab_C_ are the absorbances of treatment and control, respectively.

### Larvicidal activity

The efficacy of Se-NPs in the killing of instar larvae of *Aedes albopictus* was investigated. The larvae of *A. albopictus* mosquito have been obtained from Medical Entomology Centre, Giza, Egypt. The collected larvae were kept in plastic cups filled with deionized water and preserved under laboratory conditions. A mixture of yeast and dog food (1: 1 *w/w*) was used to feed the larvae. The experiment was conducted at 70% relative humidity, 30 °C, and photoperiod 12: 12 h (dark/light) conditions. The experiment was carried out according to the standard of WHO guidelines^77^. In this method, 25 healthy larvae from each instar (I, II, III, and IV) were incubated separately in a container containing 200 mL of tap water mixed with Se-NPs concentration (50, 40, 30, 20, and 10 µg mL^–1^) for 48 h. The container containing tap water without Se-NPs was used as a control. The larvae were considered dead if they lost their ability to reach the tap water surface after disturbance of the container. The mortality percentages were calculated using the following equation:

4 $$ \text{Larvae}\,\text{mortality}\,\%  =\frac{\text{A} - \text{B}}{\text{A}} \times 100 $$

where A is the mortality in control and B is the mortality in treatment. The experiment was carried out in five replicates for each Se-NPs concentration.

#### Statistical analysis

The statistical package SPSS v17 was used to analyze the obtained data and represented by the means of three independent replicates. The *t*-test or ANOVA followed by the Tukey HSD test at *p* < 0.05 was used to measure the difference between treatments. The larvae mortality percentages were measured by probit analysis, with LC_50_ and LC_90_ calculated using Finney’s method.

### Ethics approval and consent to participate

“Our work complies with the institutional, national, and international guidelines and legislation.”

## Conclusion

Among four endophytic fungal strains that colonized garlic roots, *P. verhagenii* was selected as the best Se-NPs producer. The selected strain was identified by traditional methods as well as sequencing of ITS genes. The synthesized Se-NPs were characterized by UV–Vis spectroscopy, FT-IR, XRD, TEM, DLS, and Zeta potential analyses. Antimicrobial activity, antioxidant, *in-vitro* cytotoxicity against cancer and normal cell lines, and larvicidal activity were investigated. Data showed varied inhibition zones due to treatment of pathogenic Gram-positive bacteria, Gram-negative, and unicellular fungi with different concentrations and MIC values in the ranges of 12.5–100 µg mL^–1^. Free radical scavenging activity was investigated using the DPPH method compared with ascorbic acid. Se-NPs reveal varied DPPH-scavenging activity based on the concentrations to be in the ranges of 31–87%. In addition, Se-NPs target cancer cells, MCF7 and PC3 with low concentrations compared to normal cells, WI38 and Vero. This finding promotes their integration into cancer treatment without negligible effects on normal cells. Moreover, the as-formed Se-NPs can be used as a larvicidal agent for a biomedical insect, *A. albopictus* with mortality percentages of 51% for IV-instar larvae and 85% for I-instar larvae at a concentration of 50 µg mL^–1^. The obtained data confirmed that the endophytic fungi possess a high potential to fabricate active Se-NPs which can integrate into various sectors.

## Supplementary Information

Supplementary Information.

## Data Availability

The datasets used and/or analyzed during the current study are available from the corresponding author on reasonable request. The sequence in the current study was deposited in NCBI (GenBank) at https://www.ncbi.nlm.nih.gov/nuccore/OP471232.

## Abbreviations

Se-NPs: Selenium nanoparticles
MCF7: Human breast cancer
PC3: Prostate cancer cell
Vero: Monkey kidney epithelial cells
WI38: Human lung fibroblast
MTT: 3-(4,5-Dimethylthiazol-2-yl)-2,5-diphenyl tetrazolium bromide
DPPH: 2,2-Diphenyl-1-picrylhydrazyl
ITS: Internal transcribed spacer
DMSO: Dimethyl sulfoxide
WHO: World Health Organization
MIC: Minimum inhibitory concentration
