# Supplementary material for: Exploring the antimicrobial, antioxidant, anticancer, biocompatibility, and larvicidal activities of selenium nanoparticles fabricated by endophytic fungal strain Penicillium verhagenii
Source: Sci Rep. 2023 Jun 3;13:9054. doi: 10.1038/s41598-023-35360-9 (PMC10239444; doi:10.1038/s41598-023-35360-9)
Supplement: Supplementary file 1 — Supplementary Information. [file 41598_2023_35360_MOESM1_ESM.docx]

**Exploring the antimicrobial, antioxidant, anticancer, biocompatibility, and larvicidal activities of selenium nanoparticles fabricated by endophytic fungal strain *Penicillium verhagenii***

Abdel-Rahman A. Nassar ^1^, Ahmed M. Eid ^2^, Hossam M. Atta ^2^, Wageih S. El Naghy ^3^, Amr Fouda ^2, ⁕^

^1^ Tanta Universal Teaching Hospital, Tanta University, Egypt.

^2^ Botany and Microbiology Department, Faculty of Science, Al-Azhar University, Nasr City, Cairo 11884, Egypt

^3^ Department of Medical Microbiology and Immunology, Faculty of Medicine, Tanta University, Egypt.

^⁕^ Corresponding author: Amr Fouda, [amr_fh83@azhar.edu.eg](mailto:amr_fh83@azhar.edu.eg) (ORCID Number: [https://orcid.org/0000-0003-3840-7837](https://www.scopus.com/redirect.uri?url=https://orcid.org/0000-0003-3840-7837&authorId=57194940078&origin=AuthorProfile&orcId=0000-0003-3840-7837&category=orcidLink))


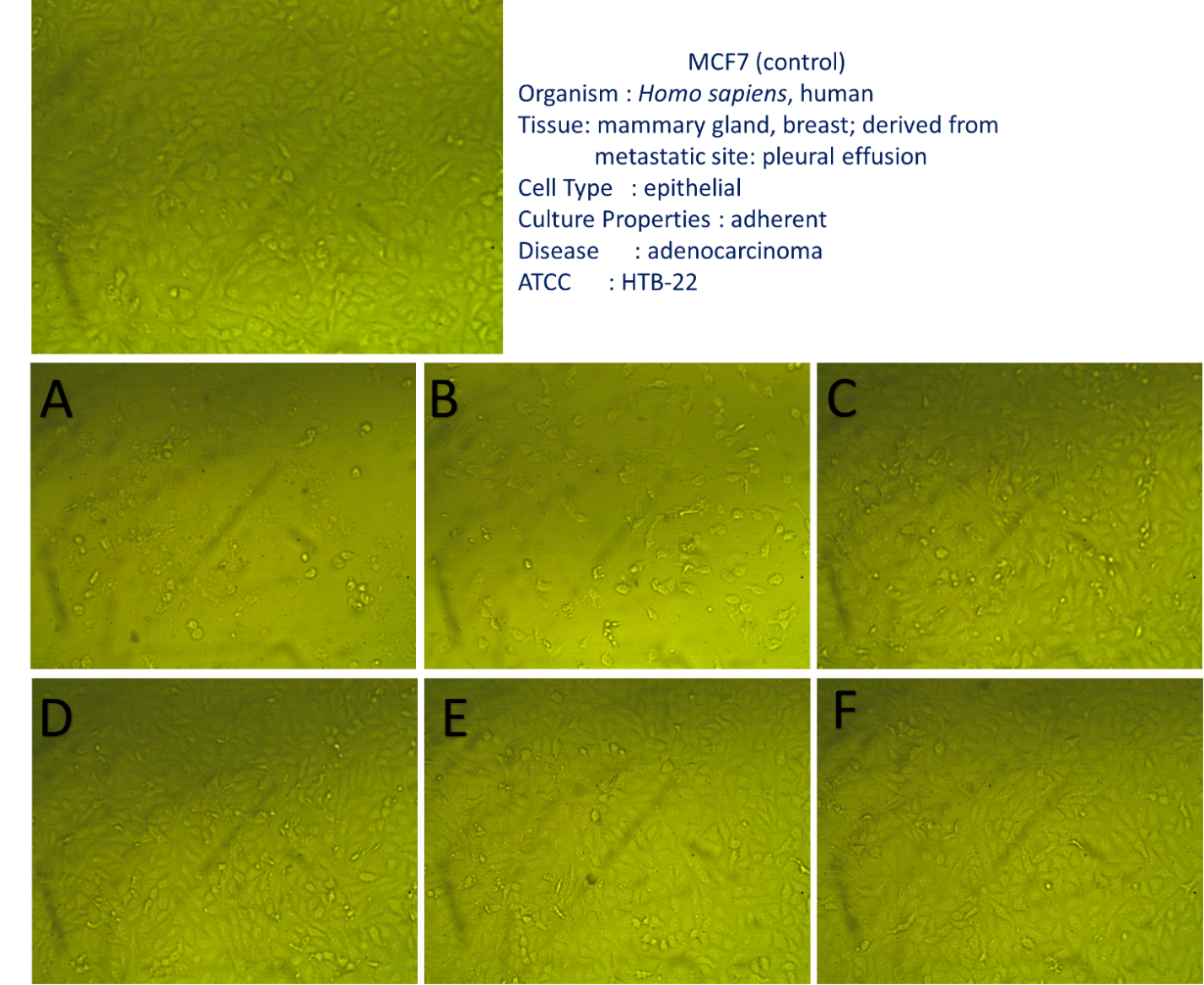


Fig. S1. Morphological changes of MCF7 after treatment with various concentrations of Se-NPs compared with control. A – F is the treatment with a concentration of 1000, 500, 250, 125, 62.5, and 31.25 µg mL^–1^ respectively.


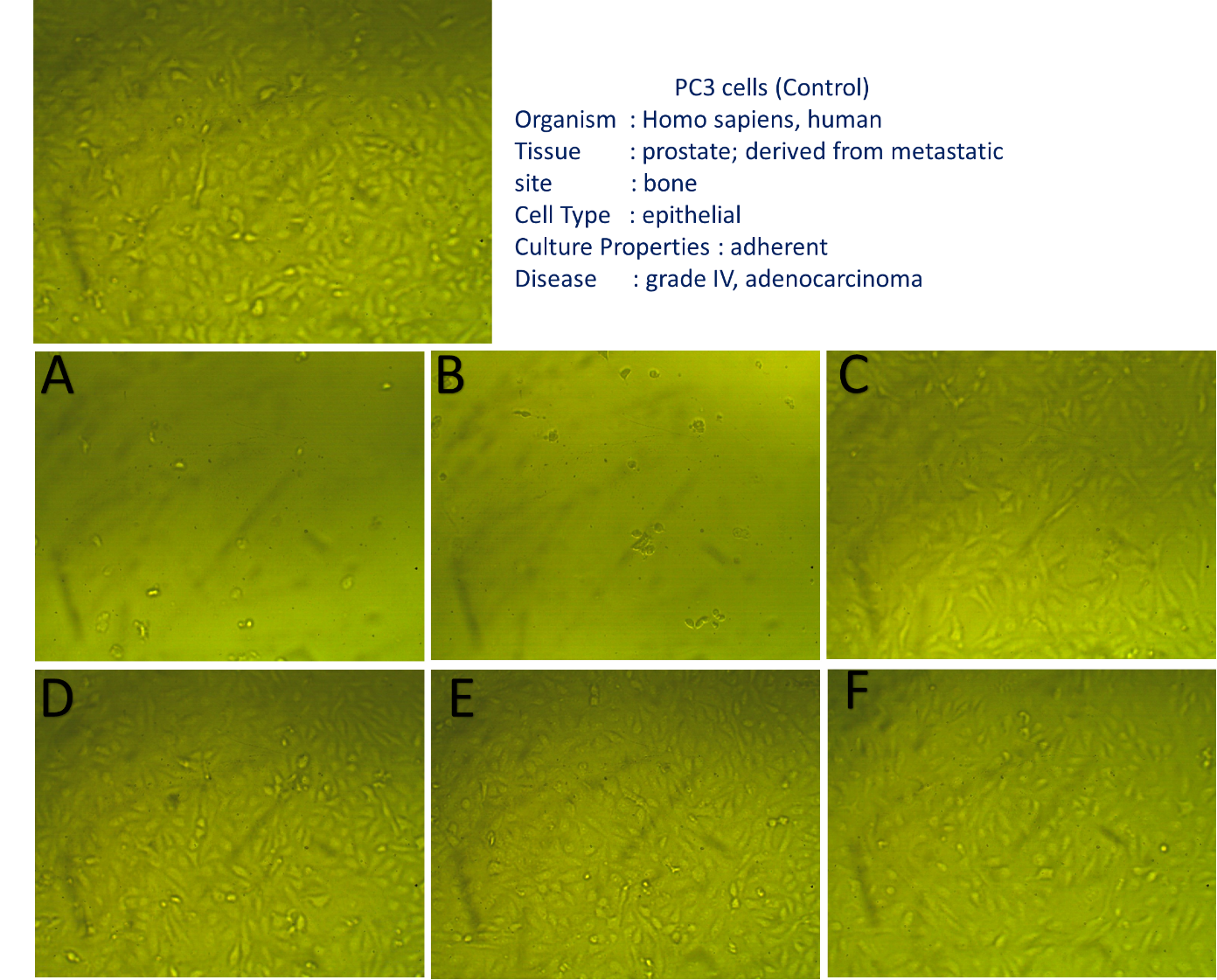


Fig. S2. Morphological changes of cancer cells PC3 after treatment with various concentrations of Se-NPs compared with control. A – F is the treatment with a concentration of 1000, 500, 250, 125, 62.5, and 31.25 µg mL^–1^ respectively.


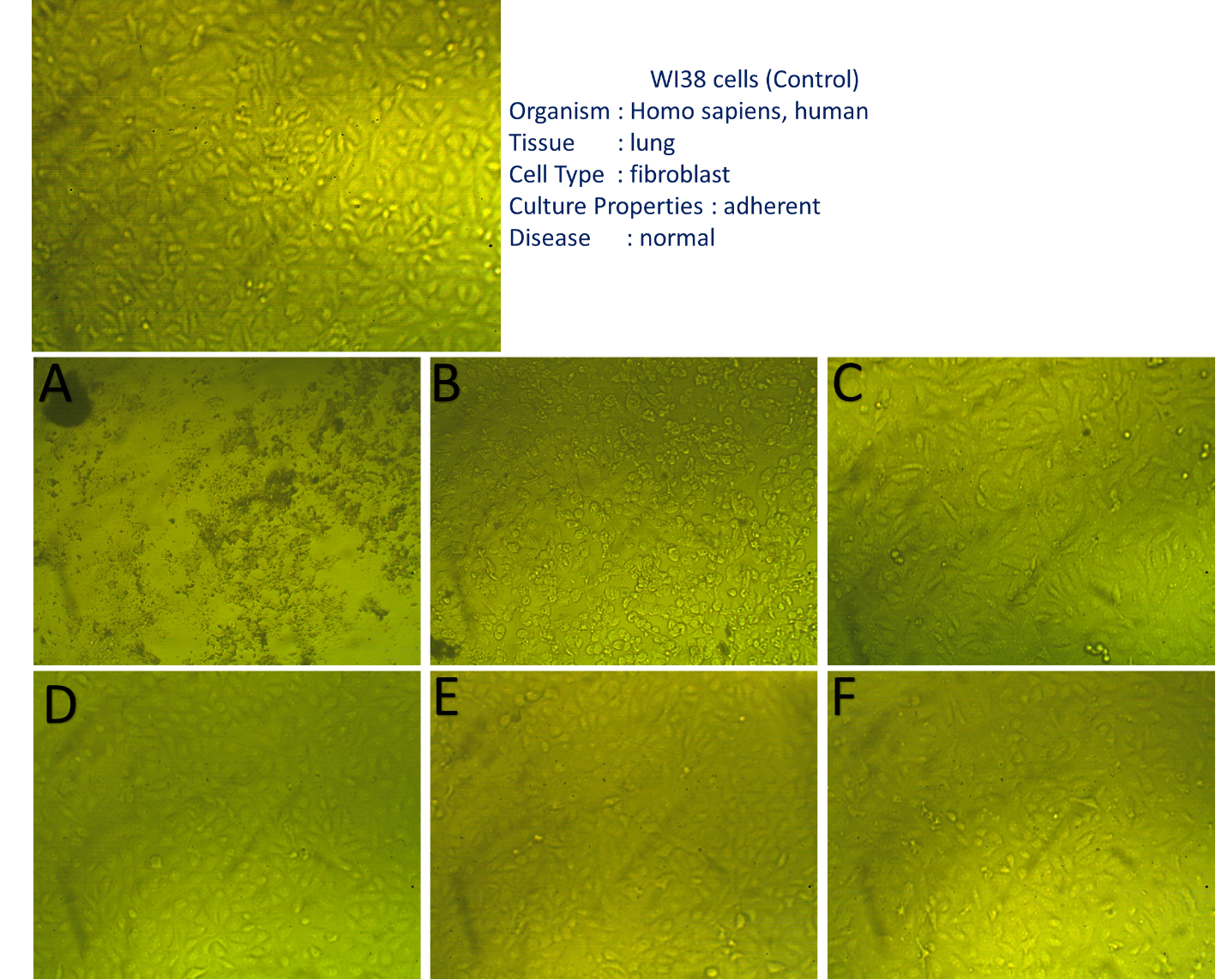


Fig. S3. Morphological changes of normal cells WI38 after treatment with various concentrations of Se-NPs compared with control. A – F is the treatment with a concentration of 1000, 500, 250, 125, 62.5, and 31.25 µg mL^–1^ respectively.


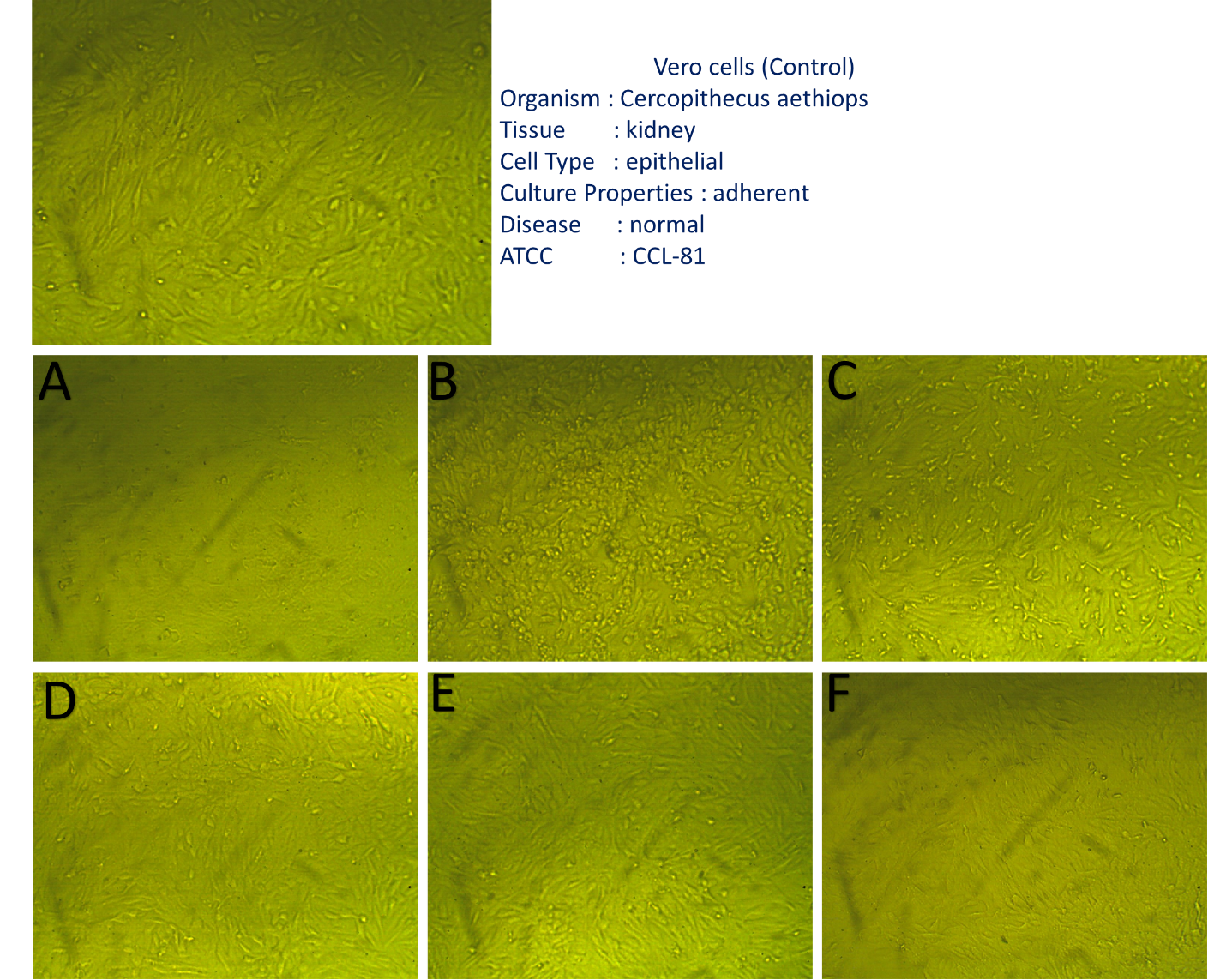


Fig. S3. Morphological changes of normal cells, Vero after treatment with various concentrations of Se-NPs compared with control. A – F is the treatment with a concentration of 1000, 500, 250, 125, 62.5, and 31.25 µg mL^–1^ respectively.
